# Very Long-Length FFT Using Multi-Resolution Piecewise-Constant Windows for Hardware-Accelerated Time–Frequency Distribution Calculations in an Ultra-Wideband Digital Receiver

**DOI:** 10.3390/s22239192

**Published:** 2022-11-26

**Authors:** Chen Wu, Janaka Elangage

**Affiliations:** Defence Research and Development Canada, Ottawa Research Centre, Ottawa, ON K1A 0Z4, Canada

**Keywords:** time-frequency analysis, short-time Fourier transform, sliding window discrete Fourier transform, blocking fast Fourier transform, ultra-wideband digital receiver, piecewise constant window function, spectrogram, electronic support measures

## Abstract

The hardware-accelerated time–frequency distribution calculation is one of the commonly used methods to analyze and present the information from intercepted radio frequency signals in modern ultra-wideband digital receiver (DRX) designs. In this paper, we introduce the piecewise constant window blocking FFT (PCW-BFFT) method. The purpose of this work is to show the generation of spectrograms (formed by a number of spectrum lines) using a very large number of samples (*N*) in an FFT frame for each spectrum line calculation. In the PCW-BFFT, the *N* samples are grouped into *K* consecutive time slots, and each slot has *M* number of samples. As soon as the *M* samples in the current time slot are available from a high-speed analog-to-digital convertor (ADC), the frequency information will be obtained using *K M*-point FFTs. Since each time the FFT frame hops one time slot for the next spectrum line calculation, the frequency information obtained from a time slot will be reused in many spectrum line calculations, as long as these spectrum lines share those samples in the time slot. Although the use of the time domain PCW introduces spikes in the frequency spectrum of the window, the levels of those spikes are still much lower than the first side lobe level of a rectangular window. Using a Gaussian window as an example, the highest spike level can be lower than the main lobe level by at least 38 dB. The PCW-BFFT method allows a DRX to produce multiple spectrograms concurrently with different analysis window widths when the time domain samples become available continuously from the ADC. This paper presents the detailed derivation process of the PCW-BFFT method and demonstrates the use of the method with simulation results. The hardware implementation process will be reported in another paper. The computer simulation results show that long signals with slowly changing frequencies over time can be depicted on the spectrograms with wide analysis windows, and short pulses and signals with rapidly changing instantaneous frequencies can be captured in the narrow analysis window spectrograms.

## 1. Introduction

Time–frequency distribution (TFD) methods have been studied and widely used for many decades, and many methods have been developed, such as short-time Fourier transform (STFT) [1,2], wavelet-based [3,4], Wigner–Ville distribution (WVD) [5], Gabor expansion [6,7], Zak transform [8,9], and many more methods [10]. These methods are used in many areas and some examples are given in [11,12,13]. A comprehensive review of the TFD methods and their applications can be found in [14].

In the modern ultra-wideband digital receiver (DRX) design, the use of TFD to study the intercepted radio frequency signals is one of the important approaches for information presentation, data analysis, and feature extraction, especially for specific emitter identification purposes [15]. Many methods have been used to produce signal TFD in DRX applications, such as the Choi–Williams distribution (CWD) [16,17,18], WVD [19,20], quadrature mirror filter bank (QMFB) [21], continuous and discrete-wavelet transform (CWT/DWT) [22,23], and STFT [24,25,26,27,28] methods. These methods have also been developed for automatic signal intra-pulse modulation analysis and classification [28,29,30,31,32] applications in radar signal analyses.

Among these TFD methods, the FFT-based STFT is still the most widely used method in DRX designs, as it can easily leverage the hardware-based FFT to speed up the spectrogram calculations, and the Fourier spectra are often used for weak signal detection applications [18,33,34]. However, the main disadvantages of STFT used in emitter signal feature extractions are that (1) the long signals with slowly changing frequencies can be hardly depicted with narrow analysis window spectrograms and (2) the short pulses and signals with rapidly changing instantaneous frequencies can only be poorly localized in the time domain if wide analysis windows are used. (Note that in this paper, we use “frame or long frame” to represent the time samples used in very long-length FFT; “analysis window” to stand for a window function, such as a Gaussian, Hann, or Hamming function; and “window” to denote an analysis window or a number of analysis windows cascaded to select a portion or portions of samples in a long frame for the N-point FFT. A window always has the same length as the long frame and hops with the frame.) Moreover, for a DRX with an ultra-wide instantaneous bandwidth, people have to use a very large number of samples in FFT (e.g., $N=2^{18\sim 20}$, i.e., long frame) to get a fine frequency resolution and achieve high receiver sensitivity [33,34]. The challenge is that such a long-frame FFT still cannot be directly implemented in today’s field programmable gate array (FPGA) for real-time signal detection and analyses in a DRX.

Xu et al. [35] introduced the blocking FFT (BFFT) method to improve the real-time performance of long-frame FFT in order to calculate a very long-length signal spectrum for embedded spectrum measurement devices. They also demonstrated how to implement the method on Xilinx’s ZYNQ-7000 device with hardware-accelerated parallel computing. The idea of the BFFT method is that a long N-point frame is grouped into K successive time slots for N frequency bin calculations, if the input sequence is a complex signal. Using the M (=N/K) samples in the current time slot available from a high-speed analog-to-digital convertor (ADC), the BFFT method does K independent M-point short-length FFTs, after a phase rotation factor being applied onto each of M samples. These phase rotation factors are the results of the BFFT that decimates the N frequency bins (frequency samples) by M and creates K frequency blocks. Hence, each frequency block has M frequency samples. In addition, the K M-point short-length FFTs can be parallel-processed on an FPGA. After the calculations using time slot samples, those K×M frequency components are added to the N frequency bins. The final N-bin Fourier spectrum is obtained by adding all of the frequency results for the total K time slots. The merit of the BFFT method is that instead of waiting for a large amount of N samples available in the onboard memory to perform one long-frame N-point FFT, which is still too big to be handled by today’s high-end FPGAs, the BFFT method performs K M-point short-length FFTs on an FPGA as soon as M samples are available.

Taking advantage of the BFFT processing of time samples in sequential time slots, the accumulatively increasing receiver sensitivity (AIRS) method was introduced in [34] for ultra-wideband DRX designs. Using AIRS, a DRX can not only cumulatively increase the sensitivity but can also achieve super-high receiver sensitivity when more time samples become available from an ADC. In addition, unlike traditional FFT-based DRX designs, an AIRS-based DRX can achieve both a fine time-of-arrival measurement resolution and fine frequency measurement resolution during the BFFT calculation. The time-of-arrival measurement resolution is determined by the length of the time slot, and the frequency measurement resolution is achieved by using a long-frame FFT, i.e., having a very large number of frequency bins in a given instantaneous frequency bandwidth. Currently, the upper limit of the instantaneous frequency bandwidth is determined by the ADC sampling rate.

In this paper, we introduce the following to extend the use of the BFFT method on the hardware for radio frequency signal detection and Fourier spectrogram calculations for signal feature extraction:

1. For the first time, we introduce the piecewise constant window BFFT (PCW-BFFT) method for hardware-accelerated spectrogram calculations. As with the STFT, the PCW-BFFT produces a signal spectrogram by computing the spectrum lines on a series of time domain frames. Each frame has N samples, and there are rN (0<r<1) samples shared between the adjacent frames [1]. In order to increase the time resolution and reduce the rabbit ear effect caused by the rectangular window, a zero-phase symmetrical analysis window function is used on each frame. According to the BFFT method, the samples in a frame are divided into K time slots, and M=N/K samples are contained in each time slot. In the PCW-BFFT, the frame hops by one time slot between adjacent spectrum line calculations, i.e., r=(K−1)/K. A hidden drawback of the traditional FFT-based spectrogram calculation is not effectively using the computational effort spent on the overlapping data in the following spectrum line calculations. This is because in each spectrum line calculation a full N-point FFT has to be performed. In contrast, in the PCW-BFFT, the frequency results obtained from the K M-point short-length FFTs of the current time slot can be reused in up to K following spectrum line calculations. This can greatly increase the calculation speed of the hardware.
2. In the traditional FFT-based STFT spectrogram calculations, each sample needs to multiply an analysis window value before the FFT. The problem with that is the samples in a given time slot get multiplied by different analysis window values when these samples are used in different long frames for different spectrum line calculations. In order to reuse the frequency results obtained from a time slot, the piecewise constant window (PCW) concept for the long-frame FFT is also introduced for the first time. Basically, instead of applying different analysis window values on the samples in a time slot, the PCW gives these samples a constant analysis window value that is equal to the prescribed analysis window function [36] value at the center of the time slot. Thus, for a given time slot, after K M-point short-length FFTs, the frequency results are multiplied by a window value that is determined by the time slot location in the long frame and the frame order in the overall spectrogram calculation. We note the following information:
  - The analysis window values can be pre-calculated and stored in a hardware memory;
  - The PCW needs a large number of time slots (K) in a long frame to approximate the original window;
3. Because of the use of the PCW, the same analysis window function (e.g., Gaussian function) with different analysis window widths or different analysis window functions with different widths can be pre-calculated and stored on hardware. In this way, a number of the spectrograms from a time sequence can be produced together, using the BFFT-processed samples that become available continuously from an ADC;
4. In addition, unlike the traditional FFT-based methods that use one analysis window on one frame, the PCW-BFFT can use a multi-resolution-window in two ways:
  - By cascading two or more analysis windows with different widths together in a window and applying them on a long frame to produce a multi-resolution spectrogram;
  - By cascading a few analysis windows from different functions (e.g., Gaussian, Hann, Hamming, etc.) with different widths in a window and applying them to a frame.

Again, in order to apply a multi-resolution-window, a large number of K and a very long frame are needed. In this paper, we only demonstrate cascading multiple analysis windows with the same function as in 4.a mentioned above.

The rest of the paper is organized as follow. The concept of the PCW-BFFT and the detailed derivation process are presented in the next section. Section 3 shows how to apply the PCW for spectrogram calculations. The computer simulation results are given in Section 4. The conclusions can be found in the last section.

## 2. PCW-BFFT Method

### 2.1. Basic Concept

**Figure 1:**
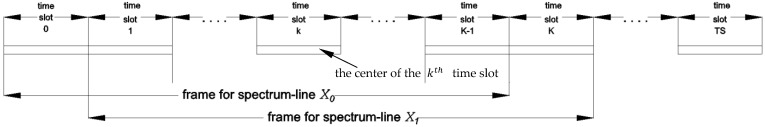
Time slots and frames used in the PCW-BFFT to calculate the spectrum lines in a spectrogram.

As mentioned before, we use “long frame or frame” to represent the time samples used in very long-length FFTs, and we use “window” to denote an analysis window or a number of analysis windows cascaded to select a portion or portions of samples in a long frame for N-point FFTs. Before we derive the PCW-BFFT method, as illustrated in Figure 1, we assume the following:

(1) The sampled data are group into TS+1 time slots;
(2) Each time slot has M samples;
(3) Each frame has N samples;
(4) N samples in a long frame are divided into K time slots, i.e., N=KM;
(5) A spectrogram is formed by a set of 1-D spectrum lines obtained from a set of consecutive long frames, so that both the frame indexes and the spectrum line indexes are the same;
(6) After each spectrum line calculation, the frame hops only one time slot for the next spectrum line calculation, and the window moves with the frame;
(7) TS is much bigger than K in order to produce a spectrogram;
(8) The indexes of time slots, frames and spectrum lines all start from zero.

Table 1 shows how a spectrogram can be calculated using the progressively available data from an ADC using the PCW-BFFT. We can observe the following:

1. The spectrum line of the $k^{th}$ frame $X_{k}\left(v\right)$ (k=0, 1, 2, …,TS) is created by an N-point FFT using samples in time slots from k to K+k−1 (see examples in Figure 1)
2. Since the frame is only shifted by one time slot between two consecutive frames, this means that $X_{k-1}\left(v\right)$ and $X_{k}\left(v\right)$ share the samples between k to K+k−2 time slots, while $X_{k-1}\left(v\right)$ uses extra samples in the ${\left(k-1\right)}^{th}$ time slot, and $X_{k}\left(v\right)$ uses extra data from the ${\left(K+k-1\right)}^{th}$ time slot;
3. The table also shows that time slot data can be reused in up to K frames. This is the main reason why the PCW-BFFT can obtain a spectrogram at almost the same speed as the samples that become available from ADC and BFFT processing.

Note that although we let the frames hop by one time slot at a time, the method developed in this paper can let the frames hop by multiple time slots as well.

As mentioned earlier, the novelty of the PCW-BFFT method is that the samples in a time slot are only used once in K M-point short-length FFTs, and the frequency results can be reused in up to K spectrum lines, as shown in Table 1. More details will be given in the next section. The PCW-BFFT tremendously reduces the time and hardware resource requirements for the TFD calculation in a DRX design, which has a high sampling rate and requires very large frequency bins for a given wide bandwidth.

### 2.2. The PCW-BFFT Method

To derive the PCW-BFFT method, we assume that N and M are to the power of 2, and the $k^{th}$ spectrum line (calculated by the $k^{th}$ frame) can be obtained using the discrete Fourier transform (DFT):

(1) $$X_{k}\left(v\right)=\overset{\infty }{\underset{n=-\infty }{\sum }}\;x\left(n\right)w\left(n-\left(\frac{N-1}{2}+kM\right)\right)W_{N}^{nv}\;\;$$

where $W_{N}=e^{-j2\pi /N}$ and w(·) is a window defined on the $k^{th}\;$ frame, whose time is centered at $\left(\frac{N-1}{2}+kM\right)\cdot T_{s}$, where $T_{s}$ is the ADC sampling interval. Since in this paper N and M are even numbers, the center of the window is always at a half time index. The value of the center of the window can always be obtained, as we have the formula of the analysis window function [36]. Considering the window/frame size N, the samples used in the $k^{th}$ frame are in [kM kM+N−1] time steps, then (1) becomes:

(2) $$X_{k}\left(v\right)=\overset{N+kM-1}{\underset{n=kM}{\sum }}\;x\left(n\right)w\left(n-\left(\frac{N-1}{2}+kM\right)\right)W_{N}^{nv}$$

First, assuming the window is rectangular with an amplitude of one (we will discuss other shapes in later sections), we have:

(3) $$X_{k}\left(v\right)=\overset{N+kM-1}{\underset{n=kM}{\sum }}\;x\left(n\right)W_{N}^{nv}$$

Following the BFFT method and using (3), the first spectrum line in Table 1 can be expressed as:

(4) $$X_{0}\left(v\right)=\overset{M-1}{\underset{n=0}{\sum }}x\left(n\right)W_{N}^{nv}+\overset{2M-1}{\underset{n=M}{\sum }}x\left(n\right)W_{N}^{nv}+\cdots +\overset{\left(k^{\prime }+1\right)M-1}{\underset{n=k^{\prime }M}{\sum }}x\left(n\right)W_{N}^{nv}+\cdots +\overset{N-1}{\underset{n=\left(K-1\right)M}{\sum }}x\left(n\right)W_{N}^{nv}$$

where $k^{\prime }$ is the index of the $k^{\prime^{th}}$ time slot. (Hereafter, we use $k^{\prime }\;$ to indicate a time slot and k to indicate a spectrum line in Table 1 or the frame to calculate the spectrum line.) The second spectrum line in Table 1 should be:

(5) $$X_{1}\left(v\right)=\overset{2M-1}{\underset{n=M}{\sum }}x\left(n\right)W_{N}^{nv}+\cdots +\overset{\left(k^{\prime }+1\right)M-1}{\underset{n=k^{\prime }M}{\sum }}x\left(n\right)W_{N}^{nv}+\ldots +\overset{N-1}{\underset{n=\left(K-1\right)M}{\sum }}x\left(n\right)W_{N}^{nv}+\overset{N+M-1}{\underset{n=KM}{\sum }}x\left(n\right)W_{N}^{nv}$$

Comparing (4) and (5), we find the following:

(1) The second to the last element in (4) are all reused in (5);
(2) The first element in (4) is not reused;
(3) The second element in (4) is only used in $\;X_{0}\left(v\right)\;$ and $\;X_{1}\left(v\right)$;
(4) After reusing the calculated frequency results from (4), the only extra effort in (5) is in calculating the last element.

Table 1 shows how the spectrum lines share a time slot and the frequency results can be reused. For example, the last element from time slot K−1 in (4) is shared in the spectrum lines from $\;X_{0}\left(v\right)\;$ to $\;X_{K-1}\left(v\right)$, as highlighted in the table.

In the same way discussed above, the $k^{th}$ spectrum line in Table 1 is:

(6) $$X_{k}\left(v\right)=\overset{\left(k+1\right)M-1}{\underset{n=kM}{\sum }}x\left(n\right)W_{N}^{nv}+\ldots +\overset{\left(k^{\prime }+1\right)M-1}{\underset{n=k^{\prime }M}{\sum }}x\left(n\right)W_{N}^{nv}+\ldots +\overset{N+kM-1}{\underset{n=\left(K+k-1\right)M}{\sum }}x\left(n\right)W_{N}^{nv}$$

Considering the $\;k^{\prime^{th}}$ element in (6) with $\;u=n-k^{\prime }M$, we have:

(7) $$\overset{\left(k^{\prime }+1\right)M-1}{\underset{n=k^{\prime }M}{\sum }}x\left(n\right)W_{N}^{nv}=\overset{M-1}{\underset{u=0}{\sum }}x\left(u+k^{\prime }M\right)W_{N}^{\left(u+k^{\prime }M\right)v}$$

We define:

(8) $$Y_{k^{\prime }}\left(v\right)=\overset{M-1}{\underset{u=0}{\sum }}x\left(u+k^{\prime }M\right)W_{N}^{\left(u+k^{\prime }M\right)v}$$

and the $k^{th}\;$ spectrum line from (6) can be written as:

(9) $$X_{k}\left(v\right)=\overset{K+k-1}{\underset{k^{\prime }=k}{\sum }}\;Y_{k^{\prime }}\left(v\right)\;\;$$

where  k=0, 1, 2,…, TS. Again, only the last element of (9) is newly calculated from the M  samples in the $\;{\left(K+k-1\right)}^{th}\;$ time slot, as the others have already been calculated and used in the previous spectrum lines.

In order to let the M samples of the $k^{\prime^{th}}$ time slot contribute to all N frequency bins in a spectrum line using K M-point FFTs, the BFFT method also divides the  N  frequency bins into K equal-length frequency blocks, and each frequency block also has M  frequency bins.

Let  v=m+Kr, where m=0, 1, 2, …, K−1 and  r=0, 1, 2, …,M−1, so that a frequency block stands for a K-fold decimation of the frequency samples  {m, m+K, m+2K, m+3K, … , m+(M−1)K},  and then (8) becomes:

(10) $$Y_{k^{\prime }}\left(m+Kr\right)=\overset{M-1}{\underset{u=0}{\sum }}x\left(u+k^{\prime }M\right)W_{N}^{m\left(u+k^{\prime }M\right)}W_{M}^{ru}$$

where $W_{N}^{k^{\prime }MKr}=1$ and KM=N are used, and (10) is an  M-point DFT. We then define:

(11) $$y\left(u+k^{\prime }M,m\right)=x\left(u+k^{\prime }M\right)\;W_{N}^{m\left(u+k^{\prime }M\right)}$$

where $\;x\left(u+k^{\prime }M\right)$ is the $k^{\prime^{th}}$ time slot sample in between $k^{\prime }M\;$ and $\;\left(k^{\prime }+1\right)M-1$ time steps, and u is the time index in the $k^{\prime^{th}}$ time slot (u=0, 1, 2, …M−1). Equation (11) denotes that as soon as those M time domain samples are available, each needs to be multiplied by a phase rotation factor ($W_{N}^{m\left(u+k^{\prime }M\right)})\;$ before being used in the spectrum calculations by the M-point short-length FFTs. The phase rotation factor is determined by the frequency block location (m) and the time step $n=\left(u+k^{\prime }M\right)$ in the overall sampling time series. The details on how to program the BFFT method are given in [34,35] and those on how to implement an FPGA can be found in [35].

## 3. Piecewise Constant Window Function

In the previous sections, we discussed the PCW-BFFT method with a rectangular window and how to reuse the frequency data obtained from a time slot shared in many frames, as shown in Table 1. To reduce FFT leakage, the STFT uses a shaped window that reduces the amplitude discontinuities at both ends of the rectangular window. In this section, we will discuss how to replace the rectangular window with other zero-phase symmetrical functions [36,37] in the PCW-BFFT. Although we use the Gaussian function as an example [38] of the analysis window in the PCW-BFFT, the same method can be applied with any other function as well.

We consider the $k^{th}\;$ spectrum line calculation using a Gaussian function, then (6) becomes:

(12) $$X_{k}\left(v\right)=\overset{K+k-1}{\underset{k^{\prime }=k}{\sum }}\;\;\;\left[\overset{\left(k^{\prime }+1\right)M-1}{\underset{n=k^{\prime }M}{\sum }}x\left(n\right)\cdot g\left(n-n_{k}\right)\cdot W_{N}^{nv}\right]\;$$

where $\;k^{\prime }\;$ is the index of a time slot and g(·) is a Gaussian function, and its maximum value is at the $n_{k}^{th}$ time step in the $\;k^{th}\;$ frame. Note that n  and $n_{k}\;$ are time indexes of the samples in the original time sequence sent from the ADC, while $\;n_{k}\;$ is always at one of the half time indexes, as  N and M are to the power of 2. If the window’s maximum value location is selected at the center of the $\;k^{th}\;$ frame, then $\;n_{k}=\frac{N-1}{2}+kM\;\left(k=0,\;1,\;2,\;\ldots TS\right)$; otherwise, the maximum value of a Gaussian function can be found at any location on a frame. More details will be given in the following discussion, when we form a multi-resolution window for a frame.

The problem occurs if we use g(·)x(·) to calculate the K M-point short-length FFTs as in (12) for a given time slot, then the frequency data from the time slot cannot be reused in the following frames. The reason is that the  g(·) of a time slot is altered in the different frames. Our solution to overcome this problem is to let the M samples of the $k^{\prime^{th}}$ time slot have a constant window value that equals the Gaussian function value at the center of time slot in the $\;k^{th}$ frame. This is the reason that the window is called the PCW, which is defined as:

(13) $$g_{k}\left(k^{\prime }\right)=g\left(\left(k^{\prime }+\frac{1}{2}\right)M-n_{k}\right)$$

Using (13) in (12), we have:

(14) $$X_{k}\left(v\right)=\overset{K+k-1}{\underset{k^{\prime }=k}{\sum }}\;g_{k}\left(k^{\prime }\right)\;\;\left[\overset{\left(k^{\prime }+1\right)M-1}{\underset{n=k^{\prime }M}{\sum }}x\left(n\right)\cdot W_{N}^{nv}\right]\;$$

Then, the frequency data obtained from a time slot in the square bracket of (14) can be reused in the following frames that share the time slot with  M  samples.

**Figure 2:**
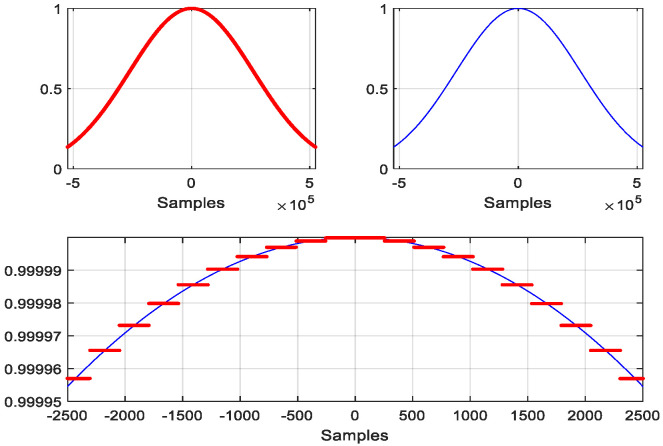
A piecewise-constant Gaussian function (**upper**-**left** in red), its corresponding original Gaussian function (**upper**-**right** in blue), and a detailed comparison in the middle portion of the Gaussian function (**lower** plot), where each short red line has 256 points $(N=2^{20},\;M=256,$ and α=2).

**Figure 3:**
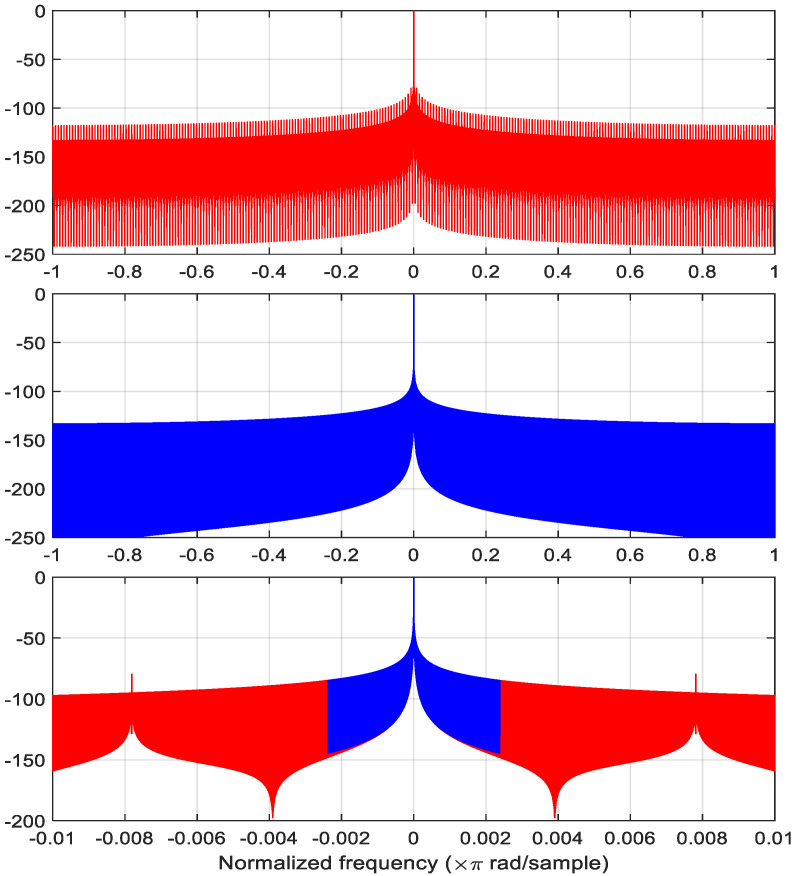
The spectra of the piecewise-constant Gaussian function (top in red), its corresponding original Gaussian function (middle in blue), and a detailed comparison in the middle portion of the spectrum (bottom) with the first spike on each side $(N=2^{20},\;M=256,\;n_{k}=0,$ and α=2).

In this study, the Gaussian function is defined as:

(15) $$g\left(n-n_{k}\right)=e^{-\frac{1}{2}{\left(\frac{2\alpha \left(n-n_{k}\right)}{N-1}\right)}^{2}}$$

where  α  is in correspondence with the standard deviation  (σ)  of a Gaussian probability density function, i.e., σ=(N−1)/(2α), and the maximum value is at the $n_{k}\;$ time step. Figure 2 shows an example of a piecewise-constant Gaussian function with $\;n_{k}=0,\;\alpha =2$, with a window size $N=2^{20},$ time slot size  M=256,  and $\;K=2^{12}$. Figure 3 shows that the frequency response of the piecewise-constant Gaussian function follows very well to that of its original Gaussian function, except for some periodic spikes. This is because of it using the piecewise constant to approximate the original Gaussian function. In this case, the frequency spacing between adjacent spikes equals $7.8125\times 10^{-3}\times \pi \;$ rad/samples, which equals  2/M. Obviously, these spikes increase the overall side lobe level of the PCW. The main concern is the differences between the levels of the main lobe and the spikes. The difference between the main lobe level and the levels of the first two spikes on each side can be defined as the dynamic range (DR) of a PCW. The DR is 85.6 dB in the case displayed Figure 3. However, when α  increases, the first spike level also increases. Table 2 gives the DR for different N, M,  and  α values. The highlighted cases will be used for the computer simulations in the next section. This shows that in the selected cases, the DRs are around 38.5 dB in the worst case.

**Figure 4:**
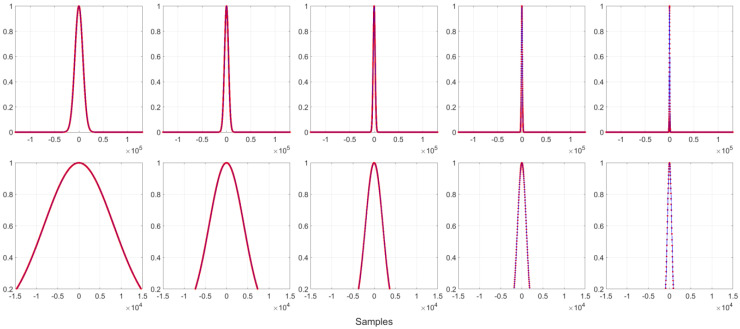
Piecewise-constant Gaussian functions with α=16, 32, 64, 128, 256  (from left to right), and $N=2^{18}$, M=64, $n_{k}=0,\;$ where the lower plots are the magnified center portions of those upper windows, while red and blue are the PCWs and their original windows, respectively.

**Figure 5:**
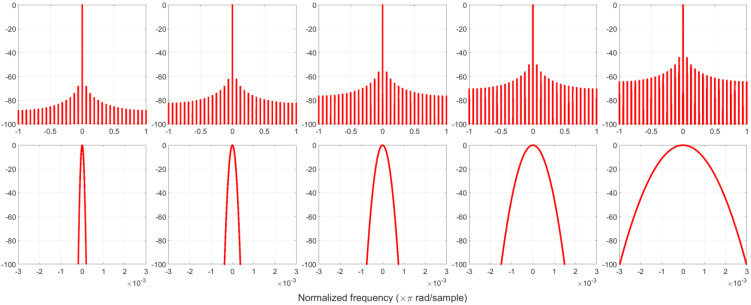
The spectra of piecewise-constant Gaussian functions in Figure 4 (upper plots), where the lower plots are the magnified center portions of the spectra. The y-axis is in dB.

Figure 4 illustrates 5 Gaussian functions with different α values. It shows that by increasing  α,  the window width decreases. The Fourier spectra of these windows are plotted in Figure 5. This shows that the power level of the spike increases as the α value increases, and the frequency spacing between the spikes is 0.03125 × π rad/samples. Figure 5 also shows that the main lobe expands as the α value increases. Although the spikes increase the overall side lobe level, we still get a 38.5 dB (Table 2) DR for the PCW in the last plot of the figure.

**Figure 6:**
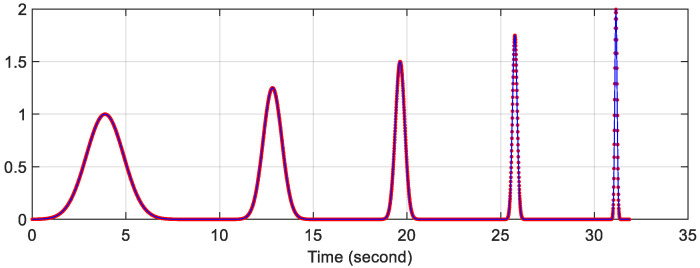
The 5 cascaded Gaussian functions shown in Figure 4. Each function is truncated at 0.0005. The red dotted line is the PCW and the blue solid line is the original window.

Figure 6 illustrates the 5 Gaussian functions ($\alpha =16,\;32,\;64,\;128,\;256,\;N=2^{18},\;M=64)\;$ cascaded to form a multi-resolution window. As this 5-resolution window is used in the first frame of the PCW-BFFT, the center (peak) locations of the 5 analysis windows are given in Table 3. The reason for the narrow widths having higher amplitudes is that the wide analysis window tends to integrate signal information from more samples than the narrow ones, which see less samples. We put more weight on the narrow ones, so that their results can be easily observed in a multi-resolution spectrogram.

In summary, in the PCW-BFFT, as soon as the frequency results of a time slot are available, before they are used in the spectrum line calculations, they will be multiplied by a window value that is determined by the frame number  k and the time slot location $\;k^{\prime }\;$ within the frame.

Based on the above discussion, the advantages of the PCW-BFFT method are as follows:

- It inherits the merit of the BFFT method, i.e., building a spectrogram, based on the readily available samples in the current time slot from the ADC, avoiding the necessity of storing a large number of samples in the hardware memory;
- It can plot a spectrogram close to the BFFT processing speed on the FPGA, as the PCW values can be stored on the hardware, and the frequency data can be reused in K spectrum lines;
- Different analysis windows can be utilized with the frequency data obtained from the current time slot to build multiple spectrograms for the data analysis and feature extraction;
- Since the BFFT has been demonstrated with an FPGA implementation [35], the PCW-BFFT should also be suitable for an FPGA implementation;
- Since there is no window applied in the AIRS method in [34], the PCW can also be applied there for weak signal detection in a DRX.

It should be noted that a PCW will have a higher side lobe level compared to its original window. As shown in Table 2, more than a 38 dB DR for the PCW still can be achieved, the spikes are still much lower than the side lobe value of the typical rectangular function, whose first side lobe level is about 13 dB lower than the main beam. Caution still has to be taken, when the PCW-BFFT is used for applications that require spectrograms with larger DRs.

The FPGA implantation of the PCW-BFFT method will be published in another paper. The computer simulated results are presented in the next section.

## 4. Simulation Results

### 4.1. Multi-Resolution Spectrogram Calculation Using the PCW-BFFT

**Figure 7:**
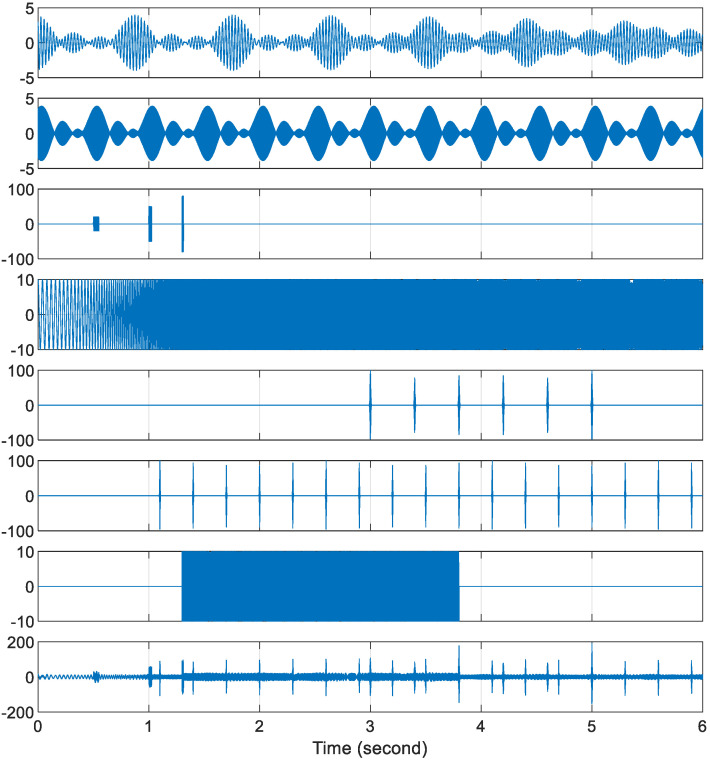
The signals used to demonstrate the PCW-BFFT method. From the top to the second last signal from the bottom, $x_{1},\;\;x_{2},\;x_{3},\;\;x_{4},\;\;x_{5},\;x_{6},$ and $x_{7}$ are presented; the details about these signals are given in the text. The bottom signal gives the sum of all 7 signals shown above.

The first testing signal used to demonstrate the PCW-BFFT method is shown in the last plot of Figure 7, which is the sum of the 7 signals shown above it. These 7 signals have different signal characteristics:

$x_{1}$**:** There are 4 sine waves in this signal, and the frequencies of these sine waves are 33.1, 34.2, 35.3, and 36.5 Hz. They appear from the beginning to the end of the signal-period. Each has an amplitude of one and a randomly picked initial phase. They can be viewed as long signals without changing their instantaneous frequencies.

$x_{2}$**:** This signal also has 4 sine waves. The frequencies are 1420, 1432, 1434, and 1436 Hz. They are also long signals. The amplitude of each sine wave is one, and their initial phases are randomly picked.

$x_{3}$**:** This signal consists of three short pulses, with the pulse widths equal to 411, 205 and 102 time steps; the amplitudes equaling 20, 50, and 80; and the carriers equaling 280, 1280, and 1907 Hz, respectively.

$x_{4}$**:** This is a quadratic chirp signal. The chirp length is 6 sec, which starts at 25 Hz and ends at 2056 Hz, with an amplitude of 10.

$x_{5}$**:** This is a 6-Gaussian-pulse train and the carrier frequency is 1777 Hz. The fractional bandwidth is 0.06, i.e., 106.62 Hz. The pulse repetition interval is 0.4 sec. The amplitude of this pulse train is 100.

$x_{6}$**:** This is another Gaussian pulse train with 17 pulses, a carrier at 1100.3 Hz, and a fractional bandwidth of 0.15, i.e., 165.05 Hz. The pulse repetition interval is 0.3 sec. The amplitude of this pulse train is also 100.

$x_{7}$**:** This is a linear chirp signal. The chirp length is 2.5 sec. It starts at 1000 Hz, finishes at 150 Hz, and its amplitude is 10.

In this simulation, the sum signals are sampled at $F_{s}=8224\;$ Hz, the frame size is $\;N=2^{18}$, and the number of samples in a time slot is  M=64, so that the number of time slots in a frame is  K=4096.

**Figure 8:**
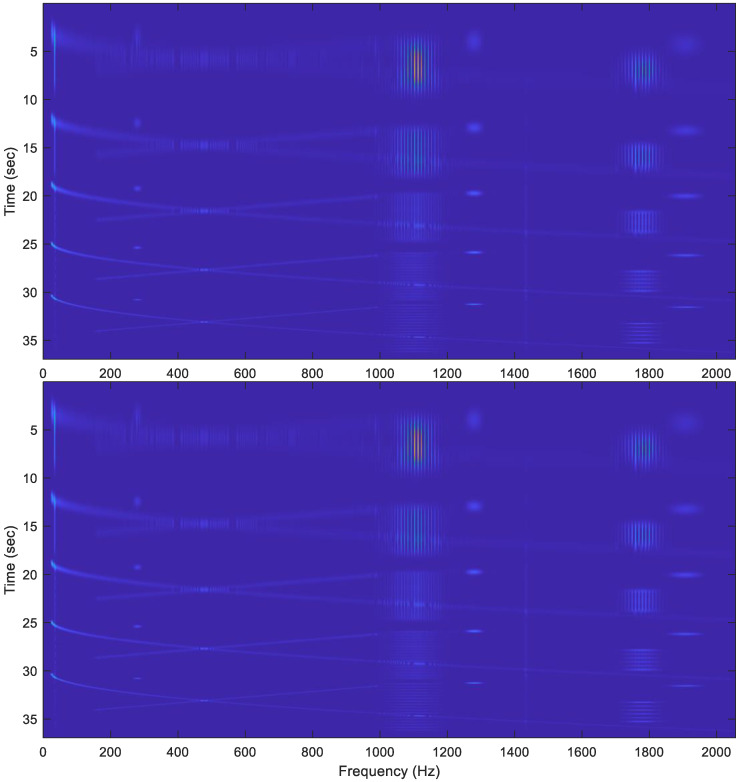
The five-resolution spectrogram of the last signal in Figure 7. The top plot is obtained using the original Gaussian functions (blue solid line in Figure 6) with the N-point FFT. The bottom plot is calculated using the piecewise-constant Gaussian function (red dotted line in Figure 6) and the PCW-BFFT method. Each plot is normalized to its maximum value.

Using the 5-resolution Gaussian function shown in Figure 6, the 5-resolution spectrogram of the testing signal is calculated and is shown in Figure 8. In the figure, two results are displayed, which are obtained as follows:

1. The original 5-resolution Gaussian functions (blue line in Figure 6) with N-point the MATLAB fft-function;
2. The piecewise-constant 5-resolution Gaussian functions (red dotted line in Figure 6) with the PCW-BFFT method.

**Figure 9:**
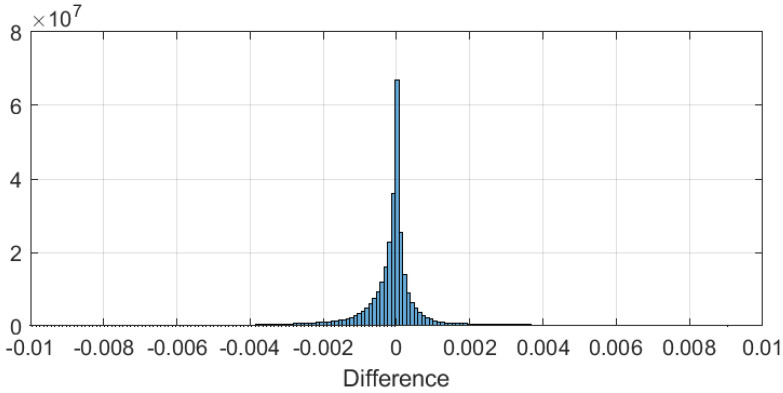
The distribution of the differences between the two spectrograms shown in Figure 8.

The errors between the two results are very small (see Figure 9). This also shows that our PCW-BFFT MATLAB code is implemented correctly.

**Figure 10:**
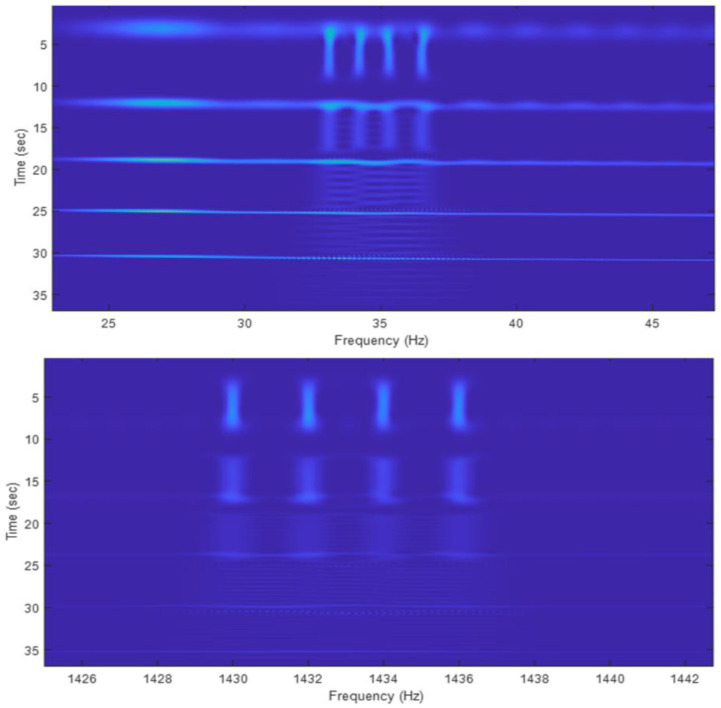
Zoom-in plots from Figure 8 at the frequencies around signal *x*_1_ (**top**) and signal *x*_2_ (**bottom**). As α increases (from **top** to **bottom** in each plot), the analysis window gets narrower, and the 4 sine wave spectra get more blurry and eventually become unidentifiable.

**Figure 11:**
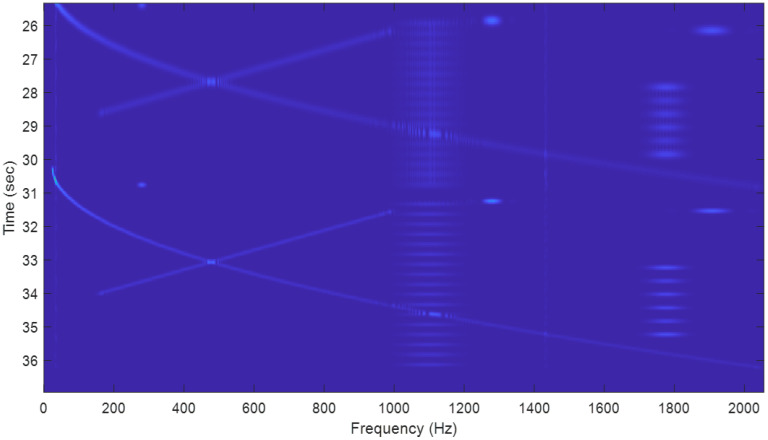
Zoom-in plots from Figure 8, looking at the last two narrow window results. The short pulses and long signals with chips are clearly captured, but these long sine waves are barely visible.

**Figure 12:**
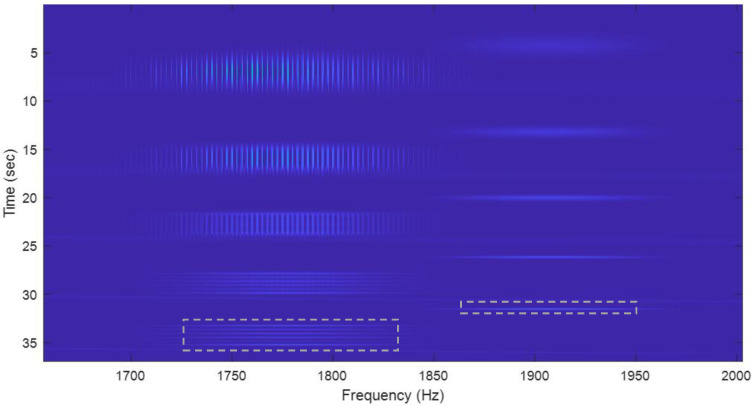
Zoom-in plots from Figure 8, looking at the frequencies at signal *x*_5_, whose carrier is at 1777 Hz, and the short pulse at 1907 Hz in *x*_3_. The short pulses are clearly captured by the narrow windows. Note that the length along the frequency axis of each line of the 2 signals (α = 256) reflects the bandwidth of the signal.

As expected, the results in Figure 8 show the following:

1. A wide window can provide a good frequency resolution for the long and slowly changing frequency signals, as shown in Figure 10.
2. A very fine frequency resolution can be observed in the figure as well;
3. A narrow window tends to capture short signals and signals with rapidly changing frequencies, as shown in Figure 11 and Figure 12.
4. Since the long-frame FFT is used in the calculation, the spectrograms have a very good frequency resolution.

### 4.2. Multiple Sprectrograms with Different Gaussian Functions Using the PCW-BFFT

**Figure 13:**
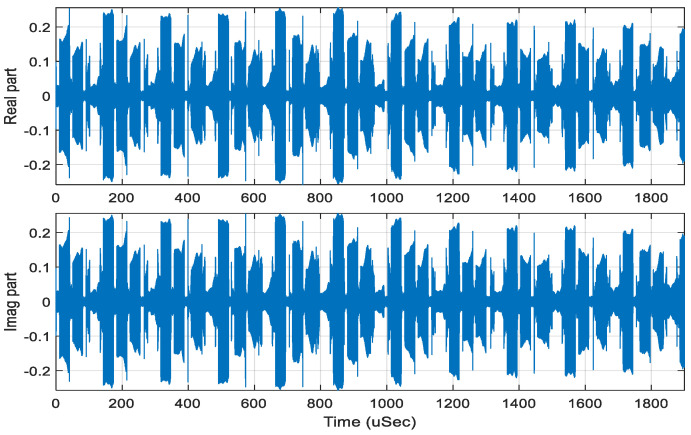
A segment of a digitized I/Q signal.

**Figure 14:**
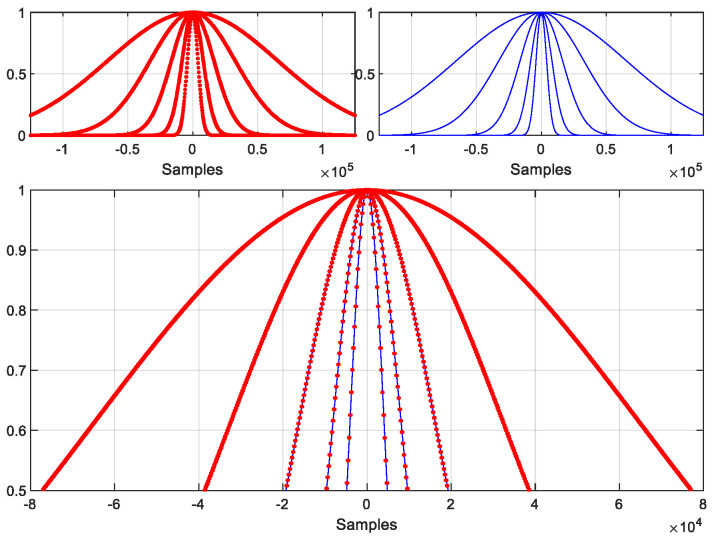
Gaussian functions with α = 8, 16, 32, 64, 128 and *N* = 2^20^, *M* = 256.

**Figure 15:**
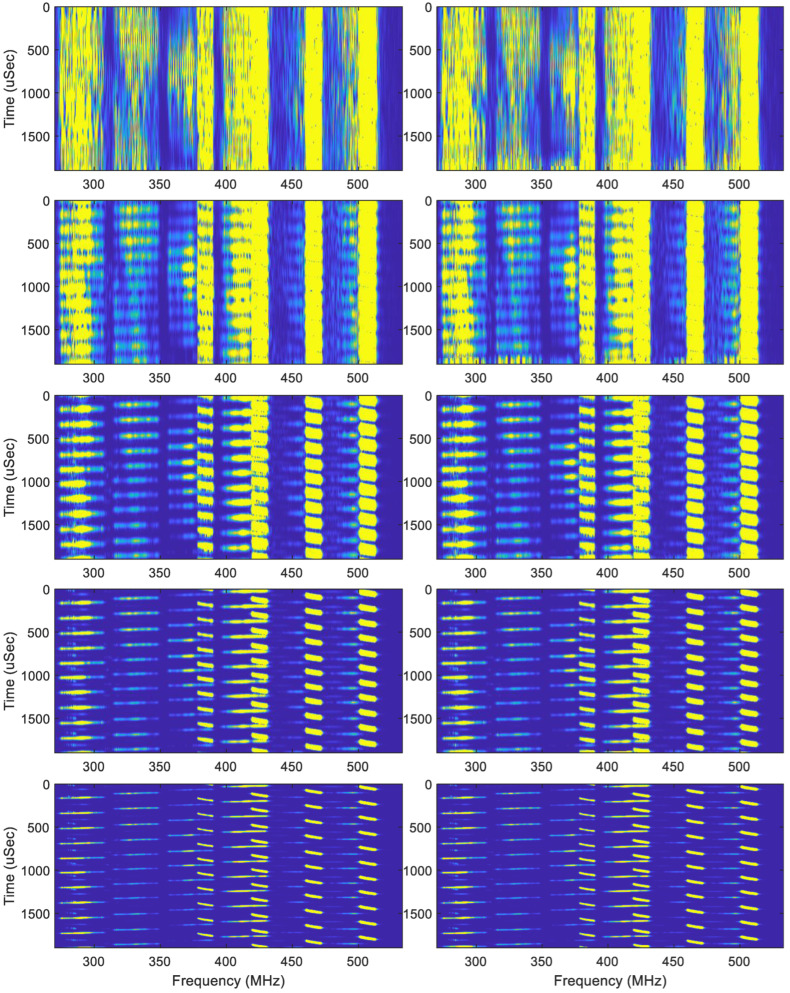
Five spectrograms of the signal in Figure 13 obtained using the five piecewise-constant Gaussian functions with different window widths shown in Figure 14. The left column results were obtained with the original Gauissian functions and the MATLAB fft-function, while the right column results were calcualted using the PCW-BFFT.

A segment of the I/Q digitized data sampled at about 530 MSamples/s from a DRX is shown in Figure 13. We use $\;N=2^{20},\;M=256$, and Gaussian functions with α=8, 16, 32, 64, 128 (as shown in Figure 14) to produce 5 spectrograms (Figure 15) simultaneously, while the BFFT performs the time-to-frequency transform. Table 2 shows that there is at least a 44 dB DR for the piecewise-constant Gaussian function with α=128.

From the sampled sequence in Figure 13, we can see that all of the signals have short durations, some have low signal-to-noise ratios, and some of them coexist in time. The results in Figure 15 show the following:

1. All of the results from the different windows have the same frequency resolution, which is determined by the $2^{20}$ bins in 530 MHz instantaneous bandwidth, which gives 505 Hz;
2. As the window width gets narrower, the focus in the time domain can be clearly observed;
3. The narrow window with a small window hopping step (483 nsec) and fine frequency resolution (505 Hz) produces spectrograms that can unveil the details of the signal characteristics in both the time and frequency domains;
4. Since a large number of frequency bins or a very small frequency bin size is used, the weak signals can be dug out from the noise;
5. A larger α has a narrower window width, which can give a good focus in the time domain. However, a window width that is too narrow may lose frequency information in the spectrogram, since there may not be enough time samples that can be used in the window. Hence, multi-resolution spectrograms allow us to study signals with different time resolutions that can show different signal characteristics.

## 5. Conclusions

This paper introduces the PCW-BFFT method suitable for calculating the TFD of a digitized time sequence with a very long-frame FFT on today’s high-end FPGA. It inherits the merits of the BFFT method of performing a very long-frame FFT using just the available samples in a time slot from a high-speed ADC, avoiding the requirement of storing a large number of samples in the hardware memory. To speed up the spectrogram calculation, the PCW allows the available frequency data obtained from the current time slot to be reused in the following K spectrum line calculations. The PCW also allows the use of multiple analysis windows with different widths to produce multiple spectrograms or a multi-resolution spectrogram, while the BFFT method continuously processes the available time slot samples from the ADC. Since the PCW is used to approximate the window, there are spikes in the PCW frequency spectrum, and the spike levels increase as the analysis window width gets narrower. However, there are still more than 38 dB between the levels of the main lobe and the spikes, which is much lower than the first side lobe level of the analysis window with a rectangular function. For a given ultra-wide instantaneous bandwidth in a DRX design, using different analysis window widths and very large samples in the time domain, the PCW-BFFT method can simultaneously produce a number of different spectrograms that have a fine frequency resolution and different time resolutions to unveil the TFD of the signals, which may have different time and frequency characteristics.

## Figures and Tables

**Table 1:** Time slots used in frames of the PCW-BFFT to calculate the spectrum lines in a spectrogram, where v is a frequency bin in a spectrum line (v=0, 1, …, N−1).

Spectrum Lines	Time Slot Index Used in Frames for Spectrum Line Calculations
$X_{0}\left(v\right)$	0, 1, 2, 3, 4, …, K−1
$X_{1}\left(v\right)$	1, 2, 3, 4, …, K−1, K
$\;X_{2}\left(v\right)$	2, 3, 4, …, K−1,K, K+1
⋯	⋯⋯⋯⋯
$X_{k-1}\left(v\right)$	k−1, k, k+1, k+2, k+3, …,K−1, K,K+1,K+2,…, K+k−2
$X_{k}\left(v\right)$	k, k+1, k+2, k+3, …,K−1,K,K+1,K+2, …, K+k−2 , K+k−1
$X_{k+1}\left(v\right)$	k+1, k+2, k+3, …,K−1,K,K+1,K+2, …, K+k−2 , K+k−1, K+k
$X_{k+2}\left(v\right)$	k+2, k+3, …,K−1,K,K+1,K+2, …, K+k−2 , K+k−1, K+k,K+k+1
⋯	⋯⋯⋯⋯
$X_{K-1}\left(v\right)$	K−1,K, K+1 ,K+2, …,K+k−2 , K+k−1,K+k,K+k+1…K+K−2
$X_{K}\left(v\right)$	K, K+1 ,K+2, …,K+k−2 , K+k−1,K+k,K+k+1…K+K−1
⋯	⋯⋯⋯⋯
$X_{TS-K}\left(v\right)$	TS−K to TS−1

**Table 2:** The first spike level (dB) with respect to the level of the main lobe. The highlighted rows show the values used in the examples.

α	2	4	8	16	32	64	128	256
$N=2^{18},\;M=32$	−85.7	−80.5	−74.4	−68.5	−62.4	−56.4	−50.3	−44.3
$N=2^{18},\;M=64$	−79.7	−74.5	−68.4	**−62.4**	**−56.4**	**−50.5**	**−44.5**	**−38.5**
$N=2^{18},\;M=128$	−73.7	−68.4	−62.5	−56.4	−50.4	−44.5	−38.2	−32.0
$N=2^{18},\;M=256$	−67.5	−62.4	−56.4	−50.5	−44.3	−38.2	−32.0	−25.7
$N=2^{19},\;M=32$	−91.7	−86.5	−80.5	−74.4	−68.4	−62.4	−56.4	−50.2
$N=2^{19},\;M=64$	−85.7	−80.5	−74.5	−68.5	−62.4	−56.4	−50.3	−44.3
$N=2^{19},\;M=128$	−79.6	−74.5	−68.5	−62.4	−56.4	−50.4	−44.5	−38.5
$N=2^{19},\;M=256$	−73.7	−68.5	−62.4	−56.4	−50.4	−44.3	−38.2	−32.0
$N=2^{20},\;M=32$	−97.7	−92.5	−86.5	−80.5	−74.4	−68.5	−62.4	−56.4
$N=2^{20},\;M=64$	−91.7	−86.5	−80.5	−74.4	−68.4	−62.5	−56.4	−50.0
$N=2^{20},\;M=128$	−85.7	−80.5	−74.5	−68.5	−62.4	−56.5	−50.3	−44.3
$N=2^{20},\;M=256$	−79.5	−74.5	**−68.4**	**−62.4**	**−56.4**	**−50.3**	**−44.3**	−38.5

**Table 3:** The peak locations of the multi-resolution window in Figure 6.

α	16	32	64	128	256
Amplitude of each analysis window	1.00	1.25	1.50	1.57	2.00
Time in the first frame (s)	3.884	12.822	19.626	25.752	31.149

## Data Availability

All simulated data are available to the readers.

## References

[1] Welch P.D. (1967). The use of fast Fourier transform for the estimation of power spectra: A method based on time averaging over short, modified periodograms. IEEE Trans. Audio Electroacoust..

[2] Crochiere R. (1980). A weighted overlap-add method of short-time Fourier analysis/Synthesis. IEEE Trans. Acoust. Speech Signal Process..

[3] Morlet J., Arens G., Fourgeau E., Glard D. (1982). Wave propagation and sampling theory—Part I: Complex signal and scattering in multilayered media. Geophysics.

[4] Morlet J., Arens G., Fourgeau E., Giard D. (1982). Wave propagation and sampling theory—Part II: Sampling theory and complex waves. Geophysics.

[5] Wigner E. (1932). On the Quantum Correction For Thermodynamic Equilibrium. Phys. Rev..

[6] Gabor D. (1946). Theory of Communication, Part 1. J. Inst. Elect. Eng. Part III Radio Commun..

[7] Bastiaans M.J. (1994). Gabor’s signal expansion and the Zak transform. Appl. Opt..

[8] Janssen A.J.E.M. (1988). The Zak transform: A signal transform for sampled time-continuous signals. Philips J. Res..

[9] Bolcskei H., Hlawatsch F. (1997). Discrete Zak transforms, polyphase transforms, and applications. IEEE Trans. Signal Process..

[10] Poularikas A.D. (2010). Transforms and Applications Handbook.

[11] Brunet P., Rimkunas Z., Temme S. Evaluation of time-frequency analysis methods and their practical applications. Audio Engineering Society. Proceedings of the Audio Engineering Society Convention 123.

[12] Moca V.V., Bârzan H., Nagy-Dăbâcan A., Mureșan R.C. (2021). Time-frequency super-resolution with superlets. Nat. Commun..

[13] Morales S., Bowers M.E. (2022). Time-frequency analysis methods and their application in developmental EEG data. Dev. Cogn. Neurosci..

[14] Boashash B. (2016). Time-Frequency Signal Analysis and Processing: A Comprehensive Review.

[15] Langley L.E. Specific emitter identification (SEI) and classical parameter fusion technology. Proceedings of the WESCON’93.

[16] Zhang M., Liu L., Diao M. (2016). LPI Radar Waveform Recognition Based on Time-Frequency Distribution. Sensors.

[17] Zilberman E.R., Pace P.E. Autonomous Time-Frequency Morphological Feature Extraction Algorithm for LPI Radar Modulation Classification. Proceedings of the 2006 IEEE International Conference on Image Processing.

[18] Pace P.E. (2009). Detecting and Classifying Low Probability of Intercept Radar.

[19] Kay S., Boudreaux-Bartels G. On the optimality of the Wigner distribution for detection. Proceedings of the IEEE International Conference on Acoustics, Speech, and Signal Processing.

[20] Barbarossa S., Lemoine O. (1996). Analysis of nonlinear FM signals by pattern recognition of their time-frequency representation. IEEE Signal Process. Lett..

[21] Copeland D.B., Pace P.E. Detection and analysis of FMCW and P-4 polyphase LPI waveforms using quadrature mirror filter trees. Proceedings of the 2002 IEEE International Conference on Acoustics, Speech, and Signal Processing.

[22] Zhu B., Jin W.D. (2012). Feature Analysis of Advanced Radar Emitter Signals Based on Continuous Wavelet Transform. Applied Mechanics and Materials.

[23] Qu L.-Z., Liu H., Huang K.-J., Yang J.-A. (2021). Specific Emitter Identification Based on Multi-Domain Feature Fusion and Integrated Learning. Symmetry.

[24] Tsui J., Stephens J. (2002). Digital microwave receiver technology. IEEE Trans. Microw. Theory Tech..

[25] Gupta A., Rai A.A.B. Feature Extraction of Intra-Pulse Modulated LPI Waveforms Using STFT. Proceedings of the 2019 4th International Conference on Recent Trends on Electronics, Information, Communication & Technology (RTEICT).

[26] Lopez-Risueno G., Grajal J., Yeste-Ojeda O. (2003). Atomic decomposition-based radar complex signal interception. IEE Proc. Radar Sonar Navig..

[27] George K., Chen C.-I.H., Tsui J.B.Y. (2007). Extension of Two-Signal Spurious-Free Dynamic Range of Wideband Digital Receivers Using Kaiser Window and Compensation Method. IEEE Trans. Microw. Theory Tech..

[28] López-Risueño G., Grajal J., Sanz-Osorio Á. (2005). Digital channelized receiver based on time-frequency analysis for signal interception. IEEE Trans. Aerosp. Electron. Syst..

[29] Barbarossa S. (1995). Analysis of multicomponent LFM signals by a combined Wigner-Hough transform. IEEE Trans. Signal Process..

[30] Singh O.K., Sarada N., Srikanth T., Ravi Kishore T. Augmented identification system. Proceedings of the EW International Conference.

[31] Gulum T.O., Pace P.E., Cristi R. Extraction of polyphase radar modulation parameters using a Wigner-Ville distribu-tion-Radon transform. Proceedings of the IEEE International Conference on Acoustics, Speech, Signal Processing.

[32] Kishore T.R., Rao K.D. (2017). Automatic Intrapulse Modulation Classification of Advanced LPI Radar Waveforms. IEEE Trans. Aerosp. Electron. Syst..

[33] Tsui J.B.Y. (2010). Special Design Topics in Digital Wideband Receivers.

[34] Wu C., Tang T., Elangage J., Krishnasamy D. (2022). Accumulatively Increasing Sensitivity of Ultrawide Instantaneous Bandwidth Digital Receiver with Fine Time and Frequency Resolution for Weak Signal Detection. Electronics.

[35] Xu P., Xu F. (2019). A real-time spectral analysis method and its FPGA implementation for long-sequence signals. Meas. Sci. Technol..

[36] Wikipedia List of Window Functions. https://en.wikipedia.org/wiki/List_of_window_functions.

[37] Smith III J.O. Spectral Audio Signal Processing. https://www.dsprelated.com/freebooks/sasp/Short_Time_Fourier_Transform.html.

[38] Wikipedia Gabor Transform. https://en.wikipedia.org/wiki/Gabor_transform.

